# C3 glomerulonephritis associated with monoclonal gammopathy: a retrospective case series study from a single institute in China

**DOI:** 10.1080/0886022X.2021.1990949

**Published:** 2021-10-17

**Authors:** Xin Zhang, Xiao-Juan Yu, Dan-yang Li, Su-xia Wang, Fu-de Zhou, Ming-hui Zhao

**Affiliations:** a Renal Division, Department of Medicine, Institute of Nephrology, Peking University First Hospital, Peking University, Beijing, China; b Renal Pathology Center, Key laboratory of Renal Disease, Ministry of Health of China, Beijing, China; c Research Units of Diagnosis and Treatment of Immune-mediated Kidney Diseases, Key Laboratory of CKD Prevention and Treatment, Ministry of Education of China, Chinese Academy of Medical Sciences, Beijing, China; d Laboratory of Electron Microscopy, Pathological Centre, Peking University First Hospital, Beijing, China; e Peking-Tsinghua Center for Life Science, Beijing, China

**Keywords:** C3GN, monoclonal gammopathy, clinicopathological features, renal prognosis

## Abstract

**Objective:**

To investigate the demographic and clinicopathological features and renal outcomes of Chinese patients with C3 glomerulonephritis in the setting of monoclonal gammopathy.

**Methods:**

Patients with renal biopsy-proven C3 glomerulonephritis and detectable serum and/or urine monoclonal immunoglobulin from 2006 to 2018 in Peking University First Hospital were included, their clinical data, renal pathology type, treatment, and prognosis were collected and analyzed.

**Results:**

Nineteen patients were enrolled, accounting for 24% of C3GN patients in the study period. The mean age of onset was 55 years old and the gender ratio was 4/15 (female/male). The mean eGFR at biopsy was 49.55 ± 29.81 ml/min/1.73m^2^. The prominent clinical manifestations included nephrotic syndrome (58%), anemia (68%), microscopic hematuria and leukocyturia (58%), and hypocomplementemia (13, 68%). The IgG was the most common isotype of monoclonal Ig on immunofixation electrophoresis. Kidney biopsies revealed a relatively prominent MPGN pattern. Only two patients had direct evidence of monocle immunoglobulins acting as C3GN pathogenic factors. Two patients had concurrent TMA-like renal injuries. The median renal survival was 12 and 15 months, respectively in patients receiving conservative therapy and immunosuppressant therapy, without statistical significance. The efficacy of clone-targeted therapy needed further investigation. Plasma exchange therapy only improved one patient’s renal outcome.

**Conclusions:**

This is the first case series report of C3GN combined with monoclonal Ig in northern China. The renal prognosis of these patients is poor, and immunosuppressant therapies show no advantage over supportive therapy in renal prognosis, while the benefit of clone-targeted chemotherapy is still requiring investigation.

## Introduction

C3 glomerulopathy (C3G) is a recently defined heterogeneous group of glomerular diseases characterized by C3 dominant deposition on immunofluorescent staining, exclusion of post-infectious glomerulonephritis, and other well-defined renal diseases [1]. Based on electron microscopic examination, C3G is classified as dense deposit disease (DDD) and C3 glomerulonephritis (C3GN). The pathogenesis of C3G is due to dysregulation of complement alternative pathway (AP) activation which can be acquired (autoantibodies against complement proteins which can be polyclonal or monoclonal, for example, C3 nephritic factors, anti-complement fact H (CFH)) or genetic (e.g., CFH, C3 gene mutations) [1].

Monoclonal gammopathy, often associated with renal disorder, consists of a heterogeneous group of diseases characterized by the abnormal clonal proliferation of Ig-producing B-lymphocytes or plasma cells, including classic malignancies such as multiple myeloma and Waldenström macroglobulinemia; and the premalignant plasma cell dyscrasia termed MGUS (monoclonal gammopathy of undetermined significance) [2]. The terminology MGRS (monoclonal gammopathy of renal significance) is introduced to describe the clonal proliferative disorder that produces a nephrotoxic monoclonal Ig and does not meet previously defined hematological criteria for treatment of a specific malignancy [3,4]. Occasionally, C3G is accompanied by monoclonal gammopathy, which proposes that monoclonal immunoglobulins might cause kidney injury indirectly through interfering AP [5–9]. Monoclonal λ-dimer functioning as anti-CFH autoantibody has also been reported [10]. The studies describing C3G patients with monoclonal gammopathy [5,6,9,11,12] show chemotherapy could improve most patients’ outcomes. However, as far as we know, there is no study describing the characteristics of Chinese patients of C3GN with monoclonal gammopathy. In this retrospective study, we report in detail 19 Chinese patients of C3GN combined with monoclonal Ig in serum and (or) urine, we also review the clinicopathological features, complement abnormalities, treatment, and follow-up of these patients.

## Methods

### Study population

A total of 80 C3G patients in Peking University First Hospital from 2006 to 2018 were retrospectively reviewed for this study, accounting for 0.7% of the contemporaneous total renal biopsies (11438 cases). Diagnosis of C3G was assessed by immunofluorescence according to consensus recommendations, with bright diffuse predominant C3 glomerular staining (≥2+), of at least two orders of magnitude greater than any other immune reactant (i.e., Ig). Among the C3G patients, 71 received immune fixation electrophoresis (IFE) tests, and 19 (all were C3GN) had detectable serum and/or urine monoclonal immunoglobulin on IFE. Immuno-staining of IgG, IgA, IgM, and light chains on paraffin tissue after enzyme digestion was done to exclude direct monoclonal immunoglobulin deposition further.

### Clinical, laboratory, and histopathological assessment

Clinical data, including demographic information, presenting features, medical history, laboratory findings, such as serum hemoglobin, serum creatinine, proteinuria, plasma cell counting, and other prognosis-related indicators, were reviewed and collected through inpatient records. The serum/urine immunofixation electrophoresis and serum complement levels were evaluated in the central clinical lab as regular tests. The complement factor H(CFH) and CFH-antibody were assessed using ELISA methods. The detection of monoclonal antibodies against complement factor H was carried out according to Li et al. [13]. Patients were regularly followed up in our outpatient clinic, and those who could not visit the clinic were contacted through telephone. Renal biopsy was examined by routine direct immunofluorescence, light microscopy, and electron microscopy. Two pathologists (W.SX and Y.XJ) evaluated biopsies separately. Bone marrow smears and biopsies were performed to assess the patients’ hematological status. Anemia is defined using the WHO criteria as hemoglobin <130 g/L in males and <120 g/L in females [14]. A history of hypertension is defined as systolic blood pressure (SBP)≥140 mmHg and/or diastolic blood pressure (DBP) ≥90 mmHg measured at the clinic [15]. The estimated glomerular filtration rate (eGFR) was determined using the Chronic Kidney Disease Epidemiology Collaboration (EPI) study equation. Chronic kidney disease (CKD) stage is defined as KIDGO guideline states. Endpoints include death and end-stage renal disease (ESRD).

### Statistical analysis

Statistical software SPSS 22.0 (SPSS, Chicago, IL, USA) was used. Continuous data were expressed as mean ± standard deviation or median with range. Categorical variables were presented as proportions. Differences between groups were compared using the Mann–Whitney U-test or the Kruskal–Wallis test. The renal survival was established using Kaplan–Meier methods.

## Results

### Clinical and laboratory data

From 2006 to 2018, 19 C3GN patients with monoclonal immunoglobulins on serum and/or urine IFE were included in this study, which accounted for 24% of the whole C3G population. No DDD patient was detected. The baseline demographic and clinical data were presented in Table 1. The gender ratio was 4/15 (female/male), and the median age was 55 (range: 26–76) years. The most common initial symptom was edema (11 out of 19), but still, seven patients did not have overt symptoms. The median duration between disease onset and renal biopsy was seven months (range: 3 to 72 months). The median proteinuria was 5.24 g/24 h (range: 0.24–19.12g/24 h) with mean serum albumin of 28.03 ± 7.22 g/L. Eleven patients (58%) had a nephrotic syndrome. Fourteen patients (74%) had microscopic hematuria, and 11 patients (58%) had synchronous mild leukocyturia (5–10 leukocytes/HPF). The mean eGFR was 49.55 ± 29.81 mL/min/1.73m^2^ (median: 37.39; range: 8.03–113.28 mL/min/1.73m^2^). Eleven patients (58%) had baseline renal function worse than CKD stage 3 b, and two patients (#9 and #17) were on dialysis at the time of renal biopsy. Thirteen patients (68%) had anemia, and 12 patients (63%) had a history of hypertension. Only two patients (#4 and #9) had positive autoantibodies (ANA 1:80), but neither had positive ds-DNA antibody or anti-ENA antibodies. We did a cryoglobulin test in 13 patients, only two patients had positive cryoglobulins (patient #16, polyclonal IgGκ, and patient #17, polyclonal IgG and monoclonal κ), but neither patient presented with systemic symptoms (including skin purpura, arthralgia, Raynaud’s phenomenon, and peripheral neuropathy) or renal pathological features of cryoglobulin related kidney injury (intraluminal pseudothrombi, wire-loop or vasculitis on LM, or electron-dense deposits with substructures on EM). The complement and hematological evaluations were presented in Table 2. Six patients had normal serum C3 and C4. In the remained 13 patients, 10 had decreased serum C3 level with normal C4, two had reduced both serum C3 and C4, and only one had reduced C4 with normal C3. Other complement-related tests, including complement factor H (CFH), anti-CFH antibody, and C3-Nef, were carried out in 12 patients. Three patients (#3, #4, #10) had deceased CFH levels with synchronous low C3 levels. Only patient #13 showed positive anti-CFH antibody and C3-Nef, of which the anti-CFH antibody was monoclonal IgGλ. Patient #17 had monoclonal IgGλ with anti-CFH short consensus repeats (SCR) 19–20 activity. Ten patients had monoclonal IgGκ on serum and/or urine IFE, including one patient who had monoclonal IgGκ plus κ in the urine. Seven patients had monoclonal IgGλ, including one patient who had monoclonal IgGλ plus λ in the urine. One patient had monoclonal IgAκ, and one had monoclonal IgAλ. Patient#2 did not agree to a bone marrow test, and his other tests did not indicate any malignant disease. Two patients had hematological malignancy, one with multiple myeloma (#18) and the other with chronic lymphocyte leukemia (CLL) (#19). Three patients (#4, #6, and #13) had more than 5% plasma cells on bone marrow smears.

**Table 1.** Demographic and clinical evaluation of patients with C3GN and monoclonal gammopathy.

Patient No.	Age(yrs)/Gender	Initial symptom	Disease courrse (m)	Anemia	HTN	Baseline SCr (μmol/L)	Baseline eGFR (ml/min/1.73m ^2^)	UTP (g/24hr)	Urine microscopy	Alb (g/L)	Autoantibody	Cryoglobulin
**1**	46/M	Fatigue	5	No	No	95	82.37	2.08	Negative	42.7	Negative	Negative
**2**	62/M	Proteinuria	72	No	Yes	167	37.22	3.65	RBCs	28.3	Negative	NA
**3**	63/M	Gross hematuria	5	Yes	Yes	215.9	27.65	2.05	RBCs and WBCs	38.2	Negative	Negative
**4**	70/M	Edema	48	Yes	No	69.8	91	4.28	RBCs and WBCs	20	ANA 1：80	Negative
**5**	47/M	Edema	9	Yes	Yes	181.5	37.39	13.22	RBCs and WBCs	21.3	Negative	Negative
**6**	60/M	Proteinuria	6	Yes	Yes	102.1	84.68	0.72	Negative	29.6	Negative	NA
**7**	70/M	Edema	7	Yes	No	88.6	70.54	6.39	RBCs and WBCs	26.2	Negative	Negative
**8**	26/M	Edema	3	Yes	Yes	158	51.25	6.31	RBCs and WBCs	27.8	Negative	0
**9**	57/M	Edema	4	No	Yes	484.9	10.63	6.93	RBCs and WBCs	24.6	ANA 1：80	0
**10**	44/M	Microscopic hematuria	48	No	No	255.6	25.25	6.36	RBCs	20.7	Negative	NA
**11**	53/M	Edema	12	Yes	Yes	282	21.04	7.67	Negative	31.1	Negative	NA
**12**	47/M	Edema	30	Yes	No	101.2	75.77	7.28	RBCs and WBCs	24.5	Negative	Negative
**13**	76/M	Proteinuria	24	Yes	Yes	168.8	33.3	3.65	RBCs and WBCs	20.6	Negative	Negative
**14**	71/F	Edema	36	Yes	Yes	108.3	44.46	5.28	RBCs and WBCs	23.2	Negative	Negative
**15**	40/F	Edema	8	Yes	Yes	162.9	33.74	4.48	RBCs and WBCs	33	Negative	NA
**16**	41/F	Edema	48	Yes	Yes	195.1	26.93	19.03	RBCs and WBCs	20.2	Negative	polyclonal IgGκ
**17**	47/M	Elevated Scr	48	Yes	Yes	647.8	8.03	12.82	Negative	34.8	Negative	polyclonal IgG and monoclonal κ
**18**	47/M	Edema	3	No	No	60.69	113.28	0.24	Negative	36.2	Negative	NA
**19**	58/F	Proteinuria	3	No	No	83.1	67.08	1.15	RBCs	36.5	Negative	Negative

Abbreviations: WBCs: white blood cells; RBCs: red blood cells; N/A: not available; HTN: hypertension; UTP: proteinuria.

**Table 2.** Complement and hematological evaluation and kidney biopsy findings of patients with C3GN and monoclonal gammopathy.

Patient No.	C3 (g/L)	C4 (g/L)	CFH (ug/ml)	Anti-CFH antibody	C3-Nef	Serum/urine IFE	Plasma cell% on bone marrow	Pattern of renal injury	Immunofluorescopic findings	% GS glomeruli	% Crescents	% interstitial fibrosis	Electron dense deposits
**1**	0.89	0.27	NA	NA	NA	Negative / IgGκ	None	MsPGN	C3+	16	0	10	MES
**2**	0.88	0.21	NA	NA	NA	IgG λ/ IgGλ	1.5	FPGN	C3+++	46	0	45	MES,SU,SE
**3**	0.56↓	0.25	150.4↓	Negative	Negative	IgGκ / IgGκ	1.0	FPGN + TIN	C3+++	5	18	35	MES,SU,IM
**4**	0.51↓	0.06↓	187.3↓	Negative	Negative	IgGκ / IgGκ	6.0	EPGN	C3++, IgG±	6	0	10	MES,SE
**5**	0.81	0.25	608.7	Negative	Negative	IgGλ / IgGλ	None	FPGN + DN	C3+++, IgA+, IgG+, λ+	35	9	15	MES,IM,SE
**6**	0.59↓	N/A	NA	NA	NA	IgAκ / IgAκ	6.5	MsPGN	C3+++	0	0	0	MES,SU
**7**	0.59↓	0.246	452.6	Negative	Negative	IgGκ / IgGκ	3.0	MPGN	C3+++, IgA+	0	10	0	MES,SE
**8**	0.74	0.26	314.4	Negative	Negative	IgGκ / Negative	NA	MPGN	C3+++ ,IgA+, IgG+	32	27	30	MES,SU
**9**	0.39↓	0.12	252.4	Negative	Negative	IgGλ / IgGλ	2.5	FPGN + TIN	C3+++, IgA+	8	56	40	MES,SU
**10**	0.58↓	0.19	222.7↓	Negative	Negative	IgGλ / IgGλ	3	FPGN + TIN	C3+++	0	28	15	MES,SU,SE
**11**	0.57↓	0.374	547.5	Negative	Negative	IgGλ / IgGλ + λ	2.5	MPGN	C3+++, IgG±, IgM±, λ±	22	0	40	MES,SU
**12**	0.66	0.12	438.1	Negative	Negative	IgGκ / IgGκ + κ	1	MPGN	C3+++, C1q±	74	0	60	MES,SU,IM,SE
**13**	0.37↓	0.2	392.9	**Positive**	**Positive**	IgGλ / IgGλ	1.5-5.5	MPGN	C3+++, C1q±	0	17	10	MES,SU,SE,IM
**14**	0.50↓	0.26	432.2	Negative	Negative	IgGκ / IgGκ	2.0	MPGN	C3+++, IgM+	44	6	50	MES,SE,IM
**15**	0.55↓	0.18	NA	NA	NA	IgGκ / IgGκ	1.0	MPGN	C3+++, IgG±	2	36	20	MES,SU,IM
**16**	0.649	0.102↓	NA	NA	NA	IgGκ / IgGκ	2.0	MPGN with TMA	C3++, IgG±	4	12	40	MES, SU
**17**	0.575↓	0.109↓	575.7	Negative	Negative	IgGλ / IgGλ	1.0	MsPGN with TMA	C3+++, IgA+, IgM+	60	0	70	MES, IM
18	0.924	0.21	NA	NA	NA	IgAλ / λ	42.5	MsPGN with LCPT	C3+++, IgA+	0	0	0	MES, SU
19	<0.058↓	0.236	NA	NA	NA	IgGκ / IgGκ	1.5	FPGN + CLL interstitial infiltration	C3++,IgG±	0	25	10	SE

The normal range of C3 was 0.6–1.5g/L, C4 was 0.12–0.36 g/L, CFH was 247–1010.8 ug/ml in our institute.

CLL: chronic lymphocyte leukemia; DN: diabetic nephropathy; EPGN: Endocapillary proliferative glomerulonephritis; FPGN: focal proliferative glomerulonephritis; GS: globally sclerosed; IF: immunofluorescence; IG: immunoglobulin; IM: intramembranous; LCPT: light-chain proximal tubulopathy; MsPGN: mesangial proliferative glomerulonephritis; MES: mesangial; MPGN: membranoproliferative glomerulonephritis; SE: subepithelial; SU: subendothelial; TMA: thrombotic microangiopathy; TIN: tubulointerstitial nephritis.

### Pathological studies

**Figure 1.**
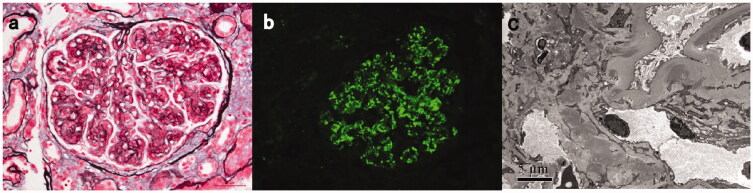
Light, immunofluorescence, and electron microscopy findings in a C3GN patient with monoclonal gammopathy (patient #15). (**A**) Light microscopy showed a membranoproliferative pattern of injury with a small fibrocellular crescent formation (Periodic acid-silver methenamine + Masson trichrome staining, ×400). **(B)** Immunofluorescence staining showed bright C3 in the mesangial and along segmental capillary walls (×400). (**C**) Electron microscopy showed electron-dense deposits in subendothelial, intramembranous and mesangial regions (×8000).

**Figure 2.**
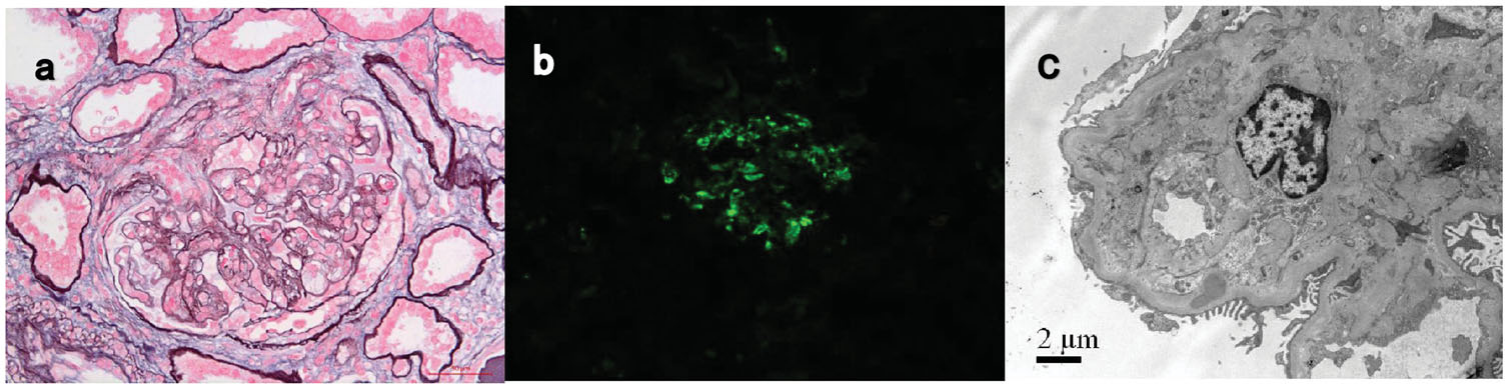
Light, immunofluorescence, and electron microscopy findings in a C3GN patient with TMA-like lesions (patient #17). (**A**) Light microscopy showed a mesangial proliferative pattern of injury with endocapillary hypercellularity and segmental sclerosis (Periodic acid-silver methenamine + Masson trichrome staining, *B* × 400). **(B)** Immunofluorescence studies showed bright C3 in the mesangial and along segmental capillary walls (×400). (**C**): Electron microscopy showed electron-dense deposits in mesangial and intramembranous regions and subendothelial edema with narrowing of the capillary lumen (×8000).

Detailed pathologic features are listed in Table 2. On light microscopy, an MPGN pattern of glomerular injury was seen in eight patients (42%) (Figure 1(a–c)), a focal proliferative pattern was seen in six patients. Four patients showed mesangial proliferative pattern and only one patient showed endocapillary proliferative glomerulonephritis. Global glomerulosclerosis and chronic tubulointerstitial lesions were prominent in 13 (68%) patients ranging from 2% to 74% and sixteen patients ranging from 10% to 70%, respectively. Nine patients had crescents with cellular/fibrocellular crescents ranging from 10% to 56%. Two patients (#16 and #17) had proliferative glomerulonephritis combined with TMA-like lesions (Figure 2(a–c)). Electron microscopy test showed electron-dense deposits in multiple areas with almost all patients; 18 patients showing mesangial deposits, 11 patients accompanied with subendothelial deposits, six patients accompanied with intra-membranous deposits, and seven patients with subepithelial deposits including one patient (#19) with just subepithelial deposits.

### Treatment and follow-up

Treatment and follow-up data are presented in Table 3. Seventeen patients were followed up for a median time of 21 months (range: 1–57 months), and two patients lost follow-up (#1 and #6) after discharge. In addition to patients #9 and #17 who were on dialysis at presentation, six patients reached ESRD in a median of 12 months (range: 5–24 months). The patients who reached ESRD showed a trend of worsening renal function at renal biopsy (37.31 [21.04–51.25] vs 67.08 [25.25–113.28] ml/min/1.7m^2^, *p* = 0.328). Three patients died in a median of 10 months (range:1 to 15 months), two of infection and one of hemorrhage. RAS inhibitors were given to 15 patients unless intolerance (hypotension or hyperkalemia). Patients #18 and #19 had hematological malignancy and received specific chemotherapy. Patient #18 received bortezomib-based chemotherapy and autologous stem cells transplantation (ASCT). He had more than a 50% decline in eGFR but stable proteinuria after ASCT. Patient #19 received COP (cyclophosphamide + oncovin + prednisone) chemotherapy. Her renal function declined more than 25% after 34 months of follow-up. The remained 17 nonmalignant patients could be divided into three groups: conservative therapy (patient #1–7), immunosuppressant therapy (patient #8–13), and chemotherapy (patient #14–17). No statistical significance was observed among the three groups on age, proteinuria, and eGFR at baseline.

**Table 3.** Treatment and kidney measures at follow up of patients with C3GN and monoclonal gammopathy.

Patient No.	Therapy	RASi	Follow-up time (months)	Death	Death Causes	Renal outcomes	Last time eGFR (ml/min/1.7m^2)	Last time UTP (g/d)
1	Supportive	No	Lost follow-up	NA	/	N/A	NA	NA
2	Supportive	Yes	43	No	/	ESRD after 12 months	NA	NA
3	Supportive	Yes	57	No	/	ESRD after 24 months	NA	NA
4	Supportive	Yes	45	No	/	Not dialysis-dependent	NA	NA
5	Supportive, PE*6	Yes	8	No	/	ESRD after 5 months	NA	NA
6	Supportive	Yes	Lost follow-up	NA	/	N/A	NA	NA
7	Supportive, PE*4	Yes	1	Yes	Sepsis	Not dialysis-dependent	NA	NA
8	Steroid alone for about 6 months	No	12	No	/	ESRD after 12 months	NA	NA
9	Steroid + CYC for about 4 months	No	15	Yes	Sepsis	Persistent ESRD	NA	NA
10	Steroid + CYC/MMF for about 12 months	Yes	45	No	/	Improved renal funcion	47.20	0.56
11	Steroid + CYC for about 6 montsh, and PE*4	Yes	13	No	/	ESRD after 6 months	NA	NA
12	Steroid + CYC/CsA/MMF for about 20 months and PE*9	Yes	27	No	/	eGFR declin*e* > 50%	14.99.	11.72
13	Steroid + CYC/MMF for about 12 months and PE*6	No	20	No	/	Improved renal function	52.51	0.24
14	PE*5 and sequential RTX 500 mg once	Yes	10	Yes	GIB	ESRD after 10 months	NA	NA
15	Steroid, CYC and Following BD*6 cycles	Yes	44	No	/	Improved renal function	68.59	0.04
16	BCD*6 cycles	Yes	9	No	/	Stable renal function	21.75	12.9
17	BCD*4 cycles	Yes	8	No	/	Persistent ESRD	10.21	2.38
18	BCD*4 cycles + ASCT	Yes	21	No	/	eGFR declin*e* > 50%	55.61	0.50
19	COP	Yes	34	No	/	eGFR declin*e* > 25%	42.98	NA

ASCT: autologous stem cells transplantation; BCD: bortezomib + cyclophosphamide + dexamethasome; BD: bortezomib + dexamethasome; COP: cyclophosphamide + Oncovin + prednisone; CYC: cyclophosphamide; CsA: cyclosporine A; GIB: gastrointestinal bleeding; MMF: mycophenolate mofetil; PE: plasma exchange; RTX: rituximab.

Patient #1–7 only received conservative therapy, including blood pressure control, use of RAS blockade, low-salt diet, and avoidance of nephrotoxic drugs. Two patients lost follow-up (#1 and #6). Despite the fully informed choice, patients #2, #3, and #4 did not agree to receive immunosuppressive therapy. Patient #5 had tuberculosis, and patient #7 had an intracranial infection, which limited the use of immunosuppressive therapy. They both received plasma exchange therapy based on supportive therapy. But patient #5 still reached ESRD after five months, and patient #7 died during hospitalization due to septic shock. Only one patient (#4) with normal renal function at baseline had a stable renal function after 45 months of follow-up. Three patients (#2, #3, and #5) reached ESRD in a median of 12 months (range: 5–24 months) with a median baseline eGFR of 37.22 mL/min/1.73m^2^ (range: 27.65–37.39 mL/min/1.73m^2^).

Patient #8–13 received either glucocorticoids alone or combined with cytotoxic agents. Patient #8 and #11 reached ESRD within 12 months. Patient #12 had more than 50% of eGFR decline but not reaching ESRD. Patient #9 died due to sepsis in 15 months after hospital discharge. Three patients (#11, #12, and #13) received plasma exchange during hospitalization due to poor response to immunosuppressive therapy, but only patient #13 showed a renal response and improved renal function. Besides patient #13, patient #10 also had improved renal function with remission of nephrotic syndrome after immunosuppressant therapy.

Patient #14–17 received chemotherapy, mainly bortezomib-based regimens (3 out of 4). Among the four patients, only patient #15 reached completed renal remission. Patient #15 received immunosuppressant therapy (prednisone and CTX) at first and once reached complete renal remission, but her nephrotic syndrome recurred two years after renal biopsy, so she turned to bortezomib-based chemotherapy. After six cycles of BD therapy, she reached complete renal remission despite persistent monoclonal IgGκ on serum IFE. Patient #14 received PE and sequential RTX 500 mg once; she never returned for more therapy, reached ESRD in 10 months, and died of gastrointestinal bleeding.

## Discussion

C3GN is one of the two renal lesions covered by the more inclusive term C3 glomerulopathy (C3G), featured by proliferative glomerulonephritis under light microscopy and predominant C3 deposit under immunofluorescent microscopy. It is distinguished from another C3G, dense deposit disease (DDD), by the location of C3 deposits, which can be subendothelial, intramembranous, or subepithelial, while the deposits are intramembranous with a ribbon-like appearance in DDD [15]. The pathogenic mechanism of C3GN and DDD are both dysregulations of complement alternative pathways, which can be autoantibodies to or mutation of alternative complement proteins or both [16–19]. The previous reports have prompted the association between C3G and monoclonal gammopathy, both MGRS and MM, especially in patients older than 50 years old [5–7,11,20]. Meri et al. [10] and Jokiranta et al. [21] found an interaction between monoclonal light-chain λ dimer and CFH protein resulting in the activation of the alternative complement pathway. In our cohort, patient #13, who was once reported by Li LL et al., was C3-Nef positive and had monoclonal IgGλ acting as anti-CFH antibody leading to MGRS-associated C3GN by interfering complement alternative pathway [13]. Patient #17, though C3-Nef and anti-CFH antibody negative, his monoclonal IgGλ were acting as anti-CFH domain 19–20 in the following functional investigation. The two patients had direct evidence of monoclonal immunoglobulins acting as C3GN pathogenic factors. But whether the presence of a monoclonal protein had a causal relationship with C3GN in the remained seventeen patients were still inconclusive. However, the proportion of monoclonal gammopathy in the patients with C3G far exceeded the expected rate in the general population according to the latest MGRS consensus, although the monoclonal Ig acting as a C3 nephritic factor or anti-factor-H antibody renal disease can be demonstrated in only about 30% of patients affected by C3 glomerulopathy, it should still be considered an MGRS-associated disorder [3].

To our knowledge, this is the first case series report describing the clinicopathologic characteristics, treatment, and renal outcomes of Chinese patients of C3GN combined with monoclonal gammopathy. The previous studies that described this disease entity are summarized in Table 4 [5–7,9,11,12]. Our study showed Chinese patients had a relatively young age of onset (median 54 years) but similar poor renal outcomes. This could be related to the relatively long interval between disease onset and diagnosis, which led to chronic renal fibrosis. We found more than half of the patients had mild leukocyturia (5–10 leukocytes/HPF). This may be due to the endocapillary proliferation (one patient) and crescents formation (nine patients). Three patients had decreased serum C4 level, including two patients with positive serum cryoglobulins, and these three patients had scanty glomerular immunoglobulins deposition, which indicated possible concurrent systemic activation of the classical complement pathway by the immune complex or lectin pathway. Renal biopsy showed a relatively predominant MPGN pattern of injury, while mesangial proliferative and focal proliferative were also present. Notably, two patients showed proliferative glomerulonephritis combined with TMA-like injury manifesting as subendothelial edema on EM and these two patients had concurrent mixed cryoglobulinemia. Whether the TMA-like injury was attributed to cryoglobulinemia or monoclonal Ig-related complement dysregulation was unclear. These two patients did not show any signs of cryoglobulinemia-related clinical manifestations or kidney pathological characteristics and showed strong C3 deposition with little immunoglobulins on IF, granular dense deposits on multiple areas which indicated that they were more consistent with C3 glomerulonephritis instead of cryoglobulinemic glomerulonephritis. Rare reports previously discussed the relationship between cryoglobulin and TMA [22,23]. The pathogenic isotype was mainly monoclonal cryoglobulinemic IgM, but our two patients had polyclonal IgG, polyclonal IgG plus monoclonal κ, respectively. Monoclonal Ig may play a vital role in complement dysregulation and the development of these two diseases. Complement factor H (CFH) deficiency and anti-CFH antibody were proved to be related with both C3G and TMA [24–26], and the different disease phenotypes were associated with the different targeted epitopes of complement factor H. Taken together, the TMA-like injury was more likely related to monoclonal Ig-related complement dysregulation.

**Table 4.** Previous reports of patients with C3G and monoclonal gammapathy.

Authors	Year	Patients, n	Age	M/F, n/n	Serum MIg, n	Haematological diagnosis, n	Treatment	Renal outcomes, n
Sethi et al. [11]	2010	10	60 (49–77)	2/8	IgGκ: 6 IgGλ: 3 IgA & IgGλ: 1	MGRS	No specific therapy	ESRD: 4; Worsen: 2; Stable: 4
Bridoux et al. [5]	2011	6	67.5 (40–74)	3/3	IgGκ: 4 IgGλ: 2	MGRS: 5 Smoldering MM: 1	No treatment: 2; Steroid: 1; Mel + Steroid: 1; Steroids + CYC: 1; BD: 1	ESRD: 5 Worsen: 1
Zand et al. [6]	2013	10	61.5 (22–69)	7/3	IgGκ: 6 IgGλ: 2 IgAλ: 1 IgMλ: 1	MGRS:9	Conservative: 4;	ESRD: 3; Stable 6; Improved: 1
Lloyd et al. [7]	2016	10	63.5 (52–90)	9/3	IgGκ: 8 IgGλ: 1 IgAκ: 1	MGRS: 5 MM: 4 Polyclonal plasmacytosis: 1	Conservative: 4; RTX + COP: 1; BD:1; Immunosuppressants: 4	ESRD: 5 Stable: 3; Improved: 2
Chauvet et al. [12]	2017	50	65 (38–82)	33/17	IgGκ: 36 IgGλ: 11 IgAκ: 1 I IgAλ: 1 λ LC only: 1	MGRS: 30 Smoldering MM: 15 Symptomatic MM: 2 CLL: 3	Conservative: 13; immunosuppressive therapy: 8; clone-directed chemotherapy: 29 (including bortezomib in 22 patients)	ESRD: 25 (9 in the chemotherapy group)
Ravindran et al. [9]	2018	36	60 (20–85)	25/11	IgG: 31 IgM: 3 IgA: 1 κ: 26 λ: 10	MGRS: 26 Smoldering MM: 2 Symptomatic MM: 5 CLL: 1 Cryoglobulinemia (type 1): 1 Cryoglobulinemia (type 2): 1	Conservative: 3; non-targeted therapy: 17; MIg-targeted therapy: 16	Improved or Stable : 44% of patient with MIg-targeted therapy and 41% of patients with non-targeted therapy.

BD: bortezomib + dexamethasome; COP: cyclophosphamide + Oncovin + prednisone; CYC: cyclophosphamide; ESRD: end-stage renal disease; MGRS: monoclonal gammopathy of renal significance; MM: multiple myeloma; MPGN: membranoproliferative glomerulonephritis; Mel: Melphalan; RTX: rituximab.

There is no currently wide-accepted treatment guideline for patients with C3GN combined with monoclonal gammopathy, the treatment options are various, and the renal outcomes are unsatisfactory. Though chemotherapy targeting abnormal B-cell clones has been introduced into the treatment of MGRS [27], the treatment efficacy and renal tolerance in old patients remained to be established. Chauvet S et al. found patients who received chemotherapy, including bortezomib, reached better renal response than those receiving conservative/immunosuppressive therapy. It was also noted that rapid hematological response appears to result in improved renal survival [12]. Sathick et al. retrospectively reviewed six highly selective patients with C3GN in the setting of monoclonal Ig and found corticosteroids alone might help restore renal function, but they may have selected patients in whom the monoclonal gammopathy may not be playing a role in the development of the C3GN [20]. In those in whom the C3GN is due to an MGRS, clone-directed therapy is required. But in China, the costly chemotherapy is not covered by health insurance unless there is clear evidence of malignant hematological disorders such as multiple myeloma or lymphoma. Given this context, the treatment options for these patients are very individualized and more reliant on the economic conditions and treatment availability than patients' hematologic status. In our study, the immunosuppressant therapies showed no advantage over supportive therapy in renal prognosis (median renal survival 12 months in conservative therapy group vs 15 months in the immunosuppressive therapy group, *p* = 0.476)). Due to the small number of patients in each group, it is impossible to derive precise conclusions regarding the efficacy of immunosuppressive or clone-targeted therapy. Plasma exchange therapy combined with immunosuppressant agents was proved effective in some C3G patients [28], and in our cohort, only one patient receiving such combination showed a renal response.

The limitations of our study are that this study is a small-size, retrospective study, the follow-up information was not so sufficient for specific analysis. Due to the natural weakness of the retrospective study, the hematological evaluation, such as the free light chain test, was absent because of economic issues, and that complement testing was not available for all patients. A prospectively planned study including total complement work up with genetics, and functional assay of monoclonal Ig on complement activation pathway is required.

## Conclusions

The prominent manifestations of Chinese patients with C3GN combined with monoclonal gammopathy are nephrotic syndrome combined with microscopic hematuria, impaired renal function, and hypocomplementemia. The renal outcome of C3GN in the setting of monoclonal Ig is poor in Chinese patients under the current immunosuppressive or conservative therapy, and the chemotherapy targeting aberrant plasma clones may be a promising treatment option needing further investigation.

## Data Availability

All data generated or analyzed during this study are included in this published article.
